# The Effect of Multiple Sclerosis on Family Planning Among Saudi Families

**DOI:** 10.7759/cureus.49353

**Published:** 2023-11-24

**Authors:** Fatimah A Albahrani, Fatima A Al Amer, Fatimah A AlSahaf, Atheer A Alhamoud, Foziah Alshamrani

**Affiliations:** 1 General Practice, King Fahad Hospital, Al-Ahsa, SAU; 2 Medicine and Surgery, King Faisal University, Al-Ahsa, SAU; 3 Neurology, King Fahad University Hospital, Al Khobar, SAU; 4 Neurology, Imam Abdulrahman Bin Faisal University, Al Khobar, SAU

**Keywords:** childbirth, pregnancy, neurology, multiple sclerosis, family planning

## Abstract

Background

Multiple sclerosis (MS) is one of the most common neurological disorders in the world, and it is the leading cause of non-traumatic disability among young adults. While genetic susceptibility plays a role in MS development, the condition is not directly hereditary. Nevertheless, MS tends to aggregate within families, with first-degree relatives of affected individuals facing a higher risk than the general population. Additionally, family planning knowledge is insufficient among MS patients. Hence, this study aimed to assess the influence of MS disease on family planning and define the factors influencing decision-making regarding family planning in multiple sclerosis patients in the Eastern province of Saudi Arabia.

Methodology

A prospective study was conducted in Eastern Province, Saudi Arabia, during the period of 2022-2023 through directly interviewing the patients using a pre-validated questionnaire. This study would improve counseling and future care plans regarding family planning during patients' visits.

Results

A total of 57 women with MS were enrolled in this study; 35 (61.4%) patients got pregnant after the onset of the disease, and 46 (97.9%) had healthy children. A total of 23 (40.4%) were previously aborted due to the disease. Only five (8.8%) diseased women experienced relapses of the disease during previous pregnancies. A total of 14 (24.6%) women reported that generally their condition improved during pregnancy and childbirth, and 12 (21.1%) had no change, while four (7%) reported that their condition generally deteriorated. Also, 15 (26.3%) had no change in their condition during the first three months after birth, while four (7%) experienced deteriorated condition. Exactly 71.2% reported that the disease mostly affected their planning for pregnancy, which was of greater extent among 24.6% and partial among 14%.

Conclusion

Multiple sclerosis affects women's decisions regarding family planning. The most reported causes among affected women included anxiety about weakness and lack of strength during childbirth, concern about possible side effects from MS drugs on the fetus (congenital malformations), worry about not being able to take care of a child due to illness and the severe impact of the disease on their health.

## Introduction

Multiple sclerosis (MS) is one of the most common neurological disorders found globally and is the leading cause of non-traumatic disability among young adults [1]. MS is a demyelinating inflammatory disease of the central nervous system that damages the nerve's myelin sheath. The loss of myelin disrupts the conduction of impulses to and from the brain, and scars appear at different times and areas of the brain and spinal cord.

Although genetic susceptibility plays a role in MS development, it is not directly hereditary [2]. MS is known to aggregate within families, with the risk for first-degree family members of individuals affected by MS being higher than in the general population [3]. Symptoms of MS include fatigue, blurred vision, limb weakness, and tingling. Some patients with MS experience a relapsing-remitting pattern, while others follow a progressive course [2].

MS relapses during pregnancy are rare, but they frequently occur in the postpartum period [4]. The first three months of postpartum are considered a high-risk period for increased clinical and MRI disease activity, then returning to the pre-pregnancy level. MS does not increase the risk of congenital disabilities, ectopic pregnancies, miscarriage, or stillbirth. Additionally, it is not associated with adverse effects on fetal development [3].

MS is two to three times more common in females than males [2]. According to a study conducted in Saudi Arabia in 2020, the prevalence of MS was highest in the central region, followed by the eastern, western, northern, and southern regions, and was higher among young females across the five regions of Saudi Arabia [5].

Generally, MS is diagnosed in between 20 and 40 years of age [2]. In the Kingdom of Saudi Arabia (KSA), a recent survey done in 2020 showed that in every four patients with MS, three were aged 40 years or less at the time of MS diagnosis [5].

As MS is likely to be diagnosed during fertile and reproductive age, this can raise concerns for the patient and their partner about the implications for family planning [6]. Concerns about MS and pregnancy include thoughts on the heredity of MS, the potential effects of MS on a pregnant woman's health, and the teratogenicity associated with disease-modifying therapy use (DMTs) [6]. Lack of education, particularly regarding treatment options during pregnancy, is a significant concern for both men and women with MS [3].

The presence of MS leads women from the Middle East to avoid pregnancy because of fears about the effect of MS on their health, MS treatments' adverse effects on pregnancy or fertility, and the limitations of fertility treatments after MS diagnosis [7]. At King Saud University Medical City (KSUMC), Saudi Arabia, a study proposed that the illness has an impact on family planning through refraining from pregnancy in 21 of 51 (41.2%), delaying pregnancy in 16 of 51 (31.4%), and having more children before disease deterioration in 6 of 51 (11.8%). Also, this study demonstrated a significant association between old age and abstinence from pregnancy [8].

In the USA, UK, France, Germany, Italy, and Spain, research was carried out in 2021 to understand family planning decisions. It was stated that 116 of 332 (35%) participants reported that their plans for having children were influenced by the disease significantly. Of these, 69/116 (59%) have changed their plans, timing, and the number of children they planned to have; 47/116 (41%) decided against having children [9].

Another study in the Argentinian provinces evaluated family planning in the last pregnancy among 82 childbearing patients after MS diagnosis. Around 56.1% had a planned pregnancy, and 43.9% of patients had an unplanned pregnancy. Interestingly, the willingness to have children did not change after the diagnosis of MS in 47.8% of women with MS. The desire to have children was significantly associated with young age, patient-determined disease steps (PDDS), disease duration of less than five years, not having children before MS diagnosis, and the family planning subject being addressed by the neurologist. Getting information from healthcare workers regarding family planning was significantly associated with a planned pregnancy [10].

Family planning knowledge is insufficient among MS patients. Furthermore, there is a lack of information and research on MS patients' knowledge of the effects of DMT on family planning [6]. The decision to maintain or switch treatment is complex and is influenced by the individual circumstances of the patient [11]. The increasing complexity of MS treatment underscores the importance of sharing patient and physician decisions and personalizing treatment options [12].

Nonetheless, identifying misconceptions and educating patients can improve the quality of life of people with MS. In addition, raising awareness among physicians to discuss this aspect enhances the medical care of females who are willing to conceive and avoid complications. It was hypothesized that multiple sclerosis significantly impacts family planning decisions among Saudi families. This study investigated the influence of MS disease on family planning amongst Saudi couples in the Eastern province of Saudi Arabia to improve counseling regarding family planning during patients' visits.

## Materials and methods

Study design and study setting

This prospective study was conducted in Eastern Province, Saudi Arabia, during the time period of 2022-2023.

Inclusion and exclusion criteria

Female multiple sclerosis patients who were aged 18 years or above and were married in Eastern Province, Saudi Arabia, were included. Multiple sclerosis patients who were males, younger than 18 years, unmarried, or out of the Eastern Province were excluded from the study.

Sample size calculation

Based on the Qualtrics sample-size calculator, 57 patients was the minimal number of MS patients, and were recruited from King Fahd University Hospital in Khobar, Kingdom of Saudi Arabia, with a confidence interval of 95% and a 5% margin of error.

Ethical considerations and IRB approval

There was no direct benefit for the patients. However, it might have an indirect benefit for them. Patients' confidentiality and the privacy of their data was the priority. Nothing that led to ethical issues was used, such as the names of the participants. Written informed consent was obtained stating the demands of the study before conducting the interview, and those who agreed to participate were enrolled. There was no risk to the participants as it was a descriptive study. This study was approved by the deanship of scientific research at King Faisal University (KFU- REC-2022-OCT-ETHICS261).

Data collection

Data were collected through direct patient interviews using a validated questionnaire that was adopted from a cross-sectional study that covered research objectives through five sections including demographics, pregnancy-related, multiple sclerosis-related, decision-making-related, and medical advice-related [8].

Data analysis

The data were collected, reviewed, and then fed to Statistical Package for Social Sciences version 21 (SPSS; IBM Inc., Armonk, New York). All statistical methods used were two-tailed with an alpha level of 0.05, considering significance if the p-value was less than or equal to 0.05. Descriptive analysis was done by prescribing frequency distribution and percentage for study variables, including women's personal data, employment, and medical history. Also, clinic-epidemiological data on multiple sclerosis, obstetric history, and its effect on the disease among women were tabulated. The impact of multiple sclerosis disease on family planning among women influences the decision to delay childbearing or have more children among women, and women's attitudes and perceptions to encourage others with MS to have children were graphed. Cross tabulation was done to show the distribution of causes for deciding to delay childbearing or have more children among women with MS by the disease's effect on their decision, and factors associated with mother's pregnancy planning decision due to MS using Pearson's Chi-squared test for significance and exact probability test for small frequency distributions.

## Results

A total of 57 women with MS were included, with ages ranging from 18 to 55 years and with a mean age of 36 ± 7.4 years. The vast majority of the study women (94.7%) were Saudi. As for education level, 33 (57.9%) were university graduates, 16 (28.1%) had a secondary level of education, and five (8.8%) had post-graduate degrees. A total of 16 (28.1%) women were unemployed due to MS, 26 (45.6%) were unemployed due to other causes, and 15 (26.3%) were employed, of which 12 (80%) were non-healthcare staff. Twenty (35.1%) women had other health problems (Table 1).

**Table 1:** Biodemographic data of study women with multiple sclerosis in the Eastern Province of Saudi Arabia MS - multiple sclerosis

Biodemographic data	N	%
Age in years		
<30	11	19.3
30-39	26	45.6
40+	20	35.1
Nationality		
Saudi	54	94.7
Non-Saudi	3	5.3
Educational level		
Below secondary	3	5.3
Secondary/ diploma	16	28.1
University graduate	33	57.9
Post-graduate	5	8.8
Employment		
Unemployed due to MS	16	28.1
Unemployed due to other causes	26	45.6
Employed	15	26.3
Job title		
Non-health care staff	12	80.0
Health care staff	3	20.0
Other diseases		
Yes	20	35.1
No	37	64.9

More than half of the study women did not know about the type of MS; 18 (31.6%) had relapsing-remitting multiple sclerosis (RRMS). Exactly 47.4% of women had partially controlled disease, while 40.4% had controlled disease. The disease symptoms onset was at the age of 18-29 years among 52.6% of the study patients, while 50.9% were diagnosed in the same age category. Only six (10.5%) patients reported that they were admitted to the ICU due to a severe attack of multiple sclerosis. As for MS' effect on women's daily life, it was moderate among 36.8%, severe among 8.8%, while 33.3% had no effect at all. A total of 42.1% had their last bout of sclerosis less than one year ago, while 14% had more than five years ago. Exactly 45.6% of the study's women visited a neurologist to follow up every four to six months, 21.1% every seven to nine months, and 17.5% every one to two years (Table 2).

**Table 2:** Clinic-epidemiological data of multiple sclerosis among diseased married women residing in the Eastern Province of Saudi Arabia RRMS - relapsing-remitting multiple sclerosis; PPMS - primary progressive multiple sclerosis; PRMS - progressive-relapsing multiple sclerosis; MS - multiple sclerosis; ICU - intensive care unit

Disease data	N	%
Type of multiple sclerosis		
RRMS	18	31.6
PPMS	2	3.5
PRMS	5	8.8
Don't know	32	56.1
Current disease status		
Totally controlled	23	40.4
Partially controlled	27	47.4
Uncontrolled	6	10.5
Don't know	1	1.8
Age at first symptoms of MS		
<18	13	22.8
18-29	30	52.6
30+	14	24.6
Age at diagnosis of MS		
<18	5	8.8
18-29	29	50.9
30+	23	40.4
Admitted to the ICU of a hospital due to a severe attack of multiple sclerosis?		
Yes	6	10.5
No	51	89.5
To what extent does MS prevent you from doing your usual activities at the present time?		
Not at all	19	33.3
Mild disability	12	21.1
Moderate disability	21	36.8
Severe disability	5	8.8
When was the last bout of sclerosis you had?		
Less than a year ago	24	42.1
1-2 years ago	14	24.6
3-4 years ago	5	8.8
4-5 years ago	6	10.5
More than five years ago	8	14.0
How often do you visit a neurologist to follow up on your case with multiple sclerosis?		
Every 1-3 months	5	8.8
Every 4-6 months	26	45.6
Every 7-9 months	12	21.1
Every 10-12 months	4	7.0
Every 1-2 years	10	17.5

Exactly 35 (61.4%) study women got pregnant after the onset of the disease, and 46 (97.9%) had a healthy child. A total of 23 (40.4%) women were previously aborted due to the disease. Only five (8.8%) diseased women experienced relapses of the disease during previous pregnancies. A total of 14 (24.6%) women reported that, generally, their condition improved during pregnancy and childbirth, and 12 (21.1%) had no change. Four (7%) reported that their condition generally deteriorated. Also, 15 (26.3%) had no change in their condition during the first three months after birth, while four (7%) experienced deteriorated condition (Table 3).

**Table 3:** Obstetric history and its effect on the disease among women with multiple sclerosis residing in the Eastern Province of Saudi Arabia MS - multiple sclerosis

Obstetric data	N	%
Did you get pregnant after the onset of the disease?		
Yes	35	61.4
No	22	38.6
Number of children		
0	10	17.5
1	10	17.5
2	13	22.8
3	13	22.8
4	11	19.3
Are your children in perfect health?		
Yes	46	97.9
No	1	2.1
Not answered	10	
History of abortion		
Yes	23	40.4
No	34	59.6
Number of abortions (N=23)		
1	12	52.2
2	6	26.1
4	1	4.3
7	1	4.3
10	3	13.0
Cause of abortion (N=23)		
Due to MS	3	13.0
Due to other cause	12	52.2
Don't know	8	34.8
Have you experienced relapses of the disease during previous pregnancies?		
Yes	5	8.8
No	52	91.2
If yes, how many times?		
1	1	25.0
2	1	25.0
3	2	50.0
Not answered	1	
How did your disease condition change during pregnancy and childbirth?		
My condition generally improved	14	24.6
My condition generally deteriorated	4	7.0
No change has occurred	12	21.1
Various changes	3	5.3
Don't know	24	42.1
How did the disease condition change in general during the first three months after birth?		
My condition generally improved	7	12.3
My condition generally deteriorated	4	7.0
No change has occurred	15	26.3
Various changes	4	7.0
Don't know	27	47.4

**Figure 1:**
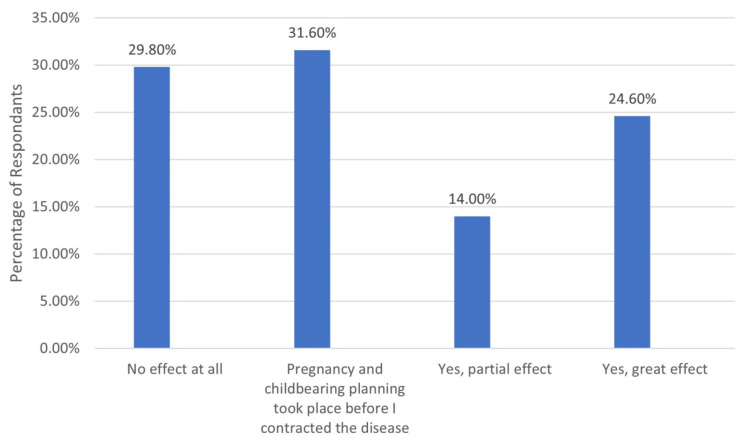
The impact of multiple sclerosis disease on family planning among women in the Eastern province of Saudi Arabia

Exactly 71.2% reported that the disease mostly affected their planning for pregnancy, which was of greater extent among 24.6% and partial among 14% of women (Figure 1).

**Figure 2:**
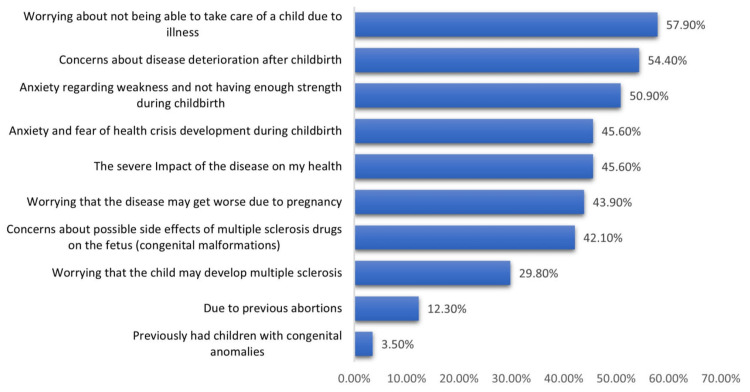
Causes deciding to delay childbearing or have more children among women with multiple sclerosis in the Eastern Province of Saudi Arabia

The most reported causes included worry about not being able to take care of a child due to illness (57.9%), concern about the deterioration of the disease after childbirth (54.4%), and anxiety about weakness and lack of strength during childbirth (50.9%) (Figure 2). 

**Figure 3:**
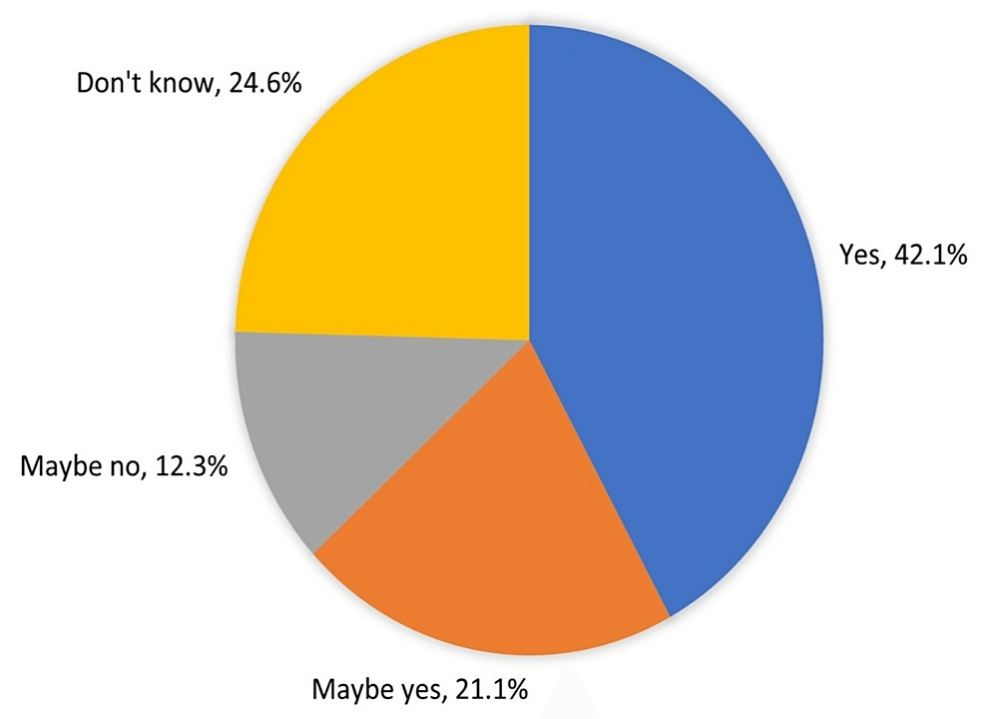
Women's attitude and perception to encourage others with MS to have children MS - multiple sclerosis

Exactly 42.1% agreed that they would encourage others to have children, while 58% of the patients were uncertain about their stance, and only 21.1% were leaning towards the encouragement of having children (Figure 3).

The most reported causes among affected women included anxiety about weakness and not having enough strength during childbirth (81.8% vs. 31.4%), concern about possible side effects from multiple sclerosis drugs on the fetus (congenital malformations) (81.8% vs. 17.1%), worrying about not being able to take care of a child due to illness (81.8% vs. 42.9%) and the severe impact of the disease on my health (63.6% vs. 34.3%) (Table 4).

**Table 4:** Distribution of causes deciding to delay childbearing or have more children among women with multiple sclerosis by the disease effect on their decision MS - multiple sclerosis

Causes influencing family planning decisions	MS affected your planning for pregnancy and childbearing?			
	Yes		No	
	N	%	N	%
The severe impact of the disease on my health	14	63.6	12	34.3
Worrying that the disease may get worse due to pregnancy	13	59.1	12	34.3
Anxiety about weakness and not having enough strength during childbirth	18	81.8	11	31.4
Anxiety about a bout of crisis during childbirth	14	63.6	12	34.3
Concern about the deterioration of the disease after childbirth	15	68.2	16	45.7
Concern about possible side effects from multiple sclerosis drugs on the fetus (congenital malformations)	18	81.8	6	17.1
Worrying about not being able to take care of a child due to illness	18	81.8	15	42.9
Worrying that the child may develop multiple sclerosis	8	36.4	9	25.7
Due to previous abortions	1	4.5	6	17.1
Previously had children with congenital anomalies	0	0.0	2	5.7

Exactly 54.5% of cases who did not get pregnant after the onset of the disease reported being affected by MS for deciding pregnancy and childbirth versus 28.6% of others who got pregnant with recorded statistical significance (p=0.049). Also, MS affected the pregnancy decision of 52.9% of women with no history of abortion compared to 17.4% of others with an abortion history (p=0.007), and all women who experienced relapses of the disease during previous pregnancies in comparison to 32.7% of others (p=0.003) (Table 5).

**Table 5:** Factors associated with mother's pregnancy planning decision due to MS P - Pearson X2 test; $ - exact probability test; * p<0.05 (significant); MS - multiple sclerosis

Factors	MS affected your planning for pregnancy and childbearing?				p-value
	Yes		No		
	N	%	N	%	
Age in years					0.625
<30	3	27.3	8	72.7	
30-39	10	38.5	16	61.5	
40+	9	45	11	55	
Education					0.997$
Below secondary	1	33.3	2	66.7	
Secondary/ diploma	6	37.5	10	62.5	
University graduate	13	39.4	20	60.6	
Post-graduate	2	40	3	60	
Employment					
Unemployed due to MS	10	62.5	6	37.5	0.039*
Unemployed due to other causes	6	23.1	20	76.9	
Employed	6	40	9	60	
Job title					
Non-health care staff	5	41.7	7	58.3	0.792$
Health care staff	1	33.3	2	66.7	
Did you get pregnant after the onset of the disease?					0.049*
Yes	10	28.6	25	71.4	
No	12	54.5	10	45.5	
Are your children in perfect health?					0.199$
Yes	17	37	29	63	
No	1	100	0	0	
History of abortion					0.007*
Yes	4	17.4	19	82.6	
No	18	52.9	16	47.1	
Cause of abortion (N=23)					0.168$ 0.003*$
Due to MS	0	0	3	100	
Due to other cause	1	8.3	11	91.7	
Don't know	3	37.5	5	62.5	
Have you experienced relapses of the disease during previous pregnancies?					
Yes	5	100	0	0	
No	17	32.7	35	67.3	
How did your disease condition change during pregnancy and childbirth?					0.618$
My condition generally improved	5	35.7	9	64.3	
My condition generally deteriorated	1	25	3	75	
No change has occurred	3	25	9	75	
Various changes	1	33.3	2	66.7	
Don't know	12	50	12	50	
How did the disease condition change in general during the first three months after birth?					0.301$
My condition generally improved	4	57.1	3	42.9	
My condition generally deteriorated	1	25	3	75	
No change has occurred	3	20	12	80	
Various changes	1	25	3	75	
Don't know	13	48.1	14	51.9	

## Discussion

Multiple sclerosis is a debilitating disease that affects the central nervous system [13]. While it is more commonly diagnosed in women in their 20s and 30s, it can affect women at any age, even during pregnancy [14, 15]. The impact of multiple sclerosis on pregnancy is an area of ongoing research, as the disease has been linked to higher rates of complications during pregnancy and childbirth [15, 16]. Pregnant women with multiple sclerosis need to work closely with their healthcare providers to ensure the best possible outcomes for both mother and baby.

The current study aimed to assess the impact of multiple sclerosis disease on family planning in Saudi families and to define the factors influencing decision-making regarding family planning in multiple sclerosis patients. The study revealed that most women ignored their MS type, but one-third had relapsing-remitting MS (RRMS). It was either totally or partially controlled among the vast majority of the study cases, which appeared mainly at the middle age (18-29 years). Research showed that RRMS was the most common type, in concordance with the current study finding [17, 18].

As for the effect of pregnancy on the disease, the current study showed that one-fourth of the study women experienced improvement during pregnancy, and one-fifth had no change, with less than 10% experiencing relapse during pregnancy. Less percent of the women experienced improved general health after birth, with less than 10% having deteriorated disease status. This was consistent with a systematic review of 22 papers that showed that there was a reduction in relapses during pregnancy and an increase in relapses in the postpartum period [19]. Also, research provided evidence that there was less relapse rate during pregnancy, particularly during the third trimester (70% decrease compared with the year before pregnancy), which matches the current study findings, but a rebound effect can occur after delivery, with increased relapses [20]. Other studies showed that there was no change in disability during pregnancy and the immediate postpartum period [21, 22].

Regarding the impact of multiple sclerosis disease on family planning among women, less than three-fourths reported that the disease mostly affected their planning for pregnancy, which was great among one-fourth and partial among others. Most reported cases included worrying about not being able to take care of a child due to illness, concern about the deterioration of the disease after childbirth, anxiety about weakness, and not having enough strength during childbirth, where all were reported among more than half of the study women. Anxiety about a bout of crisis during childbirth, the severe impact of the disease on my health, worrying that the disease might get worse due to pregnancy, and concern about possible side effects from multiple sclerosis drugs on the fetus (congenital malformations) were also reported by less than one- half of the women. Similar results were obtained by Bonavita et al. [9], as 56% of patients reported that the disease had different degrees of impact on their family planning decisions.

Further, 21% significantly changed their plans for the timing of pregnancy and the number of children, and 14% decided against having children. A lesser impact was reported by Lavorgna et al. [23], where only 29% of patients responded that the diagnosis of MS delayed their decision to become a parent. Also, a study conducted on US pregnancy rates estimated that there were significantly more pregnant women with MS in 2014 than in 2006 [24]. In France, a study assessed family planning in women with MS showed that the mean number of children per woman with MS was 1.37, compared with 1.99 children per woman in the general population, which reflected the current study's conclusion about women's pregnancy decision affected by the disease status [25].

In Saudi Arabia, Alanazy et al. [8] found that the proportion of women who abstained from or postponed pregnancy was 41.2% and 31.4%, respectively. Another study concluded that family planning should be a part of the initial conversation with a newly diagnosed patient of childbearing age. Interferons and glatiramer acetate can be continued throughout pregnancy and can be administered during breastfeeding if the benefits outweigh the risks. These DMTs might be considered for a woman with well-controlled MS who is planning a pregnancy or otherwise not using contraception [26].

Considering reasons, Houtchens et al. [24] found that concern about the ability to care for children whilst affected by MS was the main reason for delaying pregnancy. Also, a study conducted in the USA on MS patients revealed that the main MS-related reason for not becoming pregnant following MS diagnosis was the perception that symptoms would interfere with parenting (71.2%), followed by concerns of burdening their partner (50.7%) and baby inheriting MS (34.7%) [27]. Alanazy et al. [8] reported disease worsening during pregnancy, peripartum and postpartum, side effects of medications on the unborn child, and inability to care for the child. Older age was independently associated cause with the decision to abstain from pregnancy.

The empirical results reported herein should be considered in the light of some limitations. The anticipated sample size was larger than the number of patients surveyed. This was attributed mainly to difficulties reaching the targeted population of patients, as only a limited number visited healthcare facilities or organizations in the study area. Time constraints also impacted the number of patients enrolled as they can only be present a few days a month and a certain time a day. This might subject the results to biased estimates and limit generalizability. Nonetheless, data collected in this study would aid in the reduction of errors in a well-calibrated decision process regarding the patient care plan. It was recommended that more broad studies with a wider population must be done to investigate the factors that multiple sclerosis patients would consider when making decisions about their family planning.

## Conclusions

This study identified the variables impacting people with multiple sclerosis, like decision-making about family planning and the impact that the disease has on their decisions. There were 57 MS female patients ranging in age from 18 to 55 years. The disease's influence on the decisions made by women with multiple sclerosis to postpone having children or have more was distributed. The most common reasons given by women with MS were worrying about weakness and insufficient strength during childbirth. The second reported reason was worrying about possible congenital malformations caused by MS medications. Third, worrying about not being able to care for a child due to illness. Lastly, they worry about the disease's severe effects on their health status. A wider population should be included to explore the elements that multiple sclerosis patients consider when making decisions about their family planning. Therefore, it is advised to other researchers to adopt this concept and include more population to identify the precise influencing aspects of family planning in MS patients. Thereby, medical professionals can advise individuals with MS on how to enhance their quality of life.
